# MGIDI and Explainable Machine Learning for Multi-Trait Selection and Yield Drivers in Rainfed Bread Wheat

**DOI:** 10.3390/plants15182787

**Published:** 2026-09-11

**Authors:** Levent Yorulmaz, Süreyya Betül Rufaioğlu, Murat Tunç, Sibel İpekeşen, Mihriban Okur, Doğan İpekeşen, Cuma Akıncı, Mehmet Yıldırım

**Affiliations:** 1 Department of Field Crops, Faculty of Agriculture, Dicle University, Diyarbakır 21280, Türkiye; levent.yorulmaz@dicle.edu.tr (L.Y.); sibel.ipekesen@dicle.edu.tr (S.İ.); akinci@dicle.edu.tr (C.A.); 2 Department of Soil Science and Plant Nutrition, Faculty of Agriculture, Harran University, Şanlıurfa 63300, Türkiye; sureyyarufaioglu@harran.edu.tr; 3 Department of Field Crops, Faculty of Agriculture, Harran University, Şanlıurfa 63300, Türkiye; murattunc@harran.edu.tr; 4 Department of Agricultural Machinery and Technologies Engineering, Faculty of Agriculture, Dicle University, Diyarbakır 21280, Türkiye; mihriban.okur@dicle.edu.tr; 5 Department of Plant Protection, Faculty of Agriculture, Dicle University, Diyarbakır 21280, Türkiye; dogan21n@gmail.com

**Keywords:** *Triticum aestivum* L., MGIDI, multi-trait selection, explainable AI, SHAP, gradient boosting, broad-sense heritability, genetic gain, rainfed semi-arid conditions

## Abstract

Identifying superior wheat genotypes under rainfed semi-arid conditions and understanding the traits that drive yield remain challenging, and multi-trait selection indices and explainable machine learning are seldom used together. This study combined the Multi-Trait Genotype–Ideotype Distance Index (MGIDI) with explainable machine learning to evaluate 20 bread wheat (*Triticum aestivum* L.) genotypes for 14 phenological, physiological and yield-component traits in a randomised complete block design in a single rainfed season in Diyarbakır, Türkiye. Broad-sense heritability was high for all traits (H^2^ = 0.84–0.99), and the close agreement between genotypic and phenotypic coefficients of variation indicated a predominant genetic contribution to phenotypic variation. Grain yield, the number of grains per spike, and grain weight per spike showed the highest expected genetic advance (GAM = 17–22%). MGIDI-based selection of the top 25% of genotypes increased grain yield by 9.50%, with concurrent gains of 6–13% in spike length, spikelets per spike and grain weight per spike, and the relationship between MGIDI and grain yield was negative and significant (R^2^ = 0.58, *p* < 0.001). Among five learners evaluated under block-stratified GroupKFold cross-validation, Gradient Boosting performed best (CV R^2^ = 0.740; training R^2^ = 0.998; RMSE = 30.55 kg da^−1^; MAE = 24.43 kg da^−1^). SHAP analysis identified grain weight per spike, plant height and days to flowering as the main yield drivers, and these rankings were corroborated by model-independent permutation importance. Combining MGIDI with explainable machine learning allowed genotype selection and yield-determining traits to be evaluated within a single framework, and the selection decisions were biologically consistent.

## 1. Introduction

Wheat (*Triticum aestivum* L.) accounts for roughly 28% of global cereal production and holds an indispensable strategic role in nutrition and food security worldwide [1]. As a crop of critical importance for meeting basic calorie and protein requirements, it requires continuous yield improvement to satisfy the nutritional demands of a growing global population [2]. However, wheat production faces yield stagnation across a substantial part of the world’s cultivated area [3]. Agricultural production is at risk of considerable yield and quality losses due to extreme weather events driven by climate change, particularly drought, and compound drought and extreme-heat events have become more frequent and widespread across many regions in recent decades [4]. Particularly in semi-arid, rainfed-dominated regions such as the Southeastern Anatolia Region of Türkiye, yield stability shows high uncertainty in response to climatic variability, which poses a serious risk to the food supply chain [5]. Long-term meteorological analyses show that precipitation across Türkiye, including the Southeastern Anatolia Region, is highly variable and closely linked to large-scale atmospheric circulation such as the North Atlantic Oscillation [6]. For Diyarbakır in particular, trend analyses of multi-decadal climate records reveal statistically significant changes in temperature and precipitation together with pronounced drought variability [7], while intensifying summer heat stress has also been projected for the city [8]. Because the region has a Mediterranean-type precipitation regime in which rainfall is concentrated in the winter and spring months and the summer grain-filling period is largely dry, these trends heighten the risk of terminal drought during grain filling and reinforce the urgency of developing rainfed-adapted, drought-escaping wheat ideotypes for this region.

In bread wheat, grain yield is a polygenic and complex trait controlled by many genes [9]. It emerges from multifaceted interactions among phenological, physiological and yield-component traits, and selection based on grain yield alone may fail to capture the full extent of adaptive variation [10]. Modern breeding strategies have therefore shifted towards multi-trait selection approaches that simultaneously optimise interrelated traits such as yield, stress tolerance and quality [11]. The Multi-Trait Genotype–Ideotype Distance Index (MGIDI), developed in this context, removes the multicollinearity problem among traits through factor analysis, minimises the subjectivity involved in assigning economic weights, and ranks genotypes according to their Euclidean distance to an ideotype defined in the rotated factor space [12]. MGIDI also provides directly applicable decision-support outputs for breeding programmes by revealing, through standardised factor scores, the strengths and weaknesses of each genotype relative to the ideotype. This approach has performed well in identifying superior genotypes across crops such as wheat, rice and maize [13,14,15].

In recent years, artificial intelligence and machine learning (ML) techniques have contributed to an important shift in plant breeding through their capacity to model complex, non-linear relationships in agricultural systems [16]. Tree-based algorithms such as Gradient Boosting and XGBoost, in particular, often outperform conventional linear models in capturing interactions between agronomic and environmental variables and yield [17]. However, the “black-box” nature of these models is an important limitation that constrains the biological interpretability of their predictions [18]. To address this, the Shapley Additive Explanations (SHAP) method quantitatively decomposes the contribution of each variable to the model output, providing both global and local interpretability [19]. SHAP is a useful tool for identifying which traits are decisive in grain-yield formation and has been applied successfully in wheat and other cereals [20,21].

In the current literature, studies that jointly apply multi-trait selection indices such as MGIDI together with explainable machine learning approaches remain limited. Most studies treat these approaches separately and do not evaluate, within a single framework, the relationship between genotype selection and the traits that contribute to yield formation [22]. In particular, under rainfed semi-arid conditions there is a notable research gap regarding the integration of these two approaches to both identify superior genotypes and explain the biological mechanisms underlying that superiority. Standalone MGIDI, although efficient at ranking genotypes across multiple traits, does not quantify the causal weight of each individual trait on final grain yield, whereas black-box machine learning models, even when they achieve high predictive accuracy, do not deliver an ideotype-based, multi-trait balanced ranking of candidate genotypes that breeders can readily act upon. The unified framework proposed here addresses both gaps simultaneously: MGIDI performs balanced ideotype-based selection, while SHAP-explainable machine learning quantifies the biological drivers of the yield response, so that the same dataset yields both a defensible selection decision and a mechanistic interpretation of what makes the selected genotypes superior.

Accordingly, we hypothesised that (i) broad-sense heritability and genetic advance for yield-related traits would be high under rainfed semi-arid conditions; (ii) the MGIDI index would effectively distinguish balanced, high-performing genotypes showing a significant inverse relationship with grain yield; and (iii) a Gradient Boosting model supported by SHAP would identify a limited number of physiologically and agronomically consistent key traits that play a dominant role in grain-yield formation, with SHAP-based contributions consistent with model-independent permutation-importance rankings. The objectives were to characterise genotypic variation, estimate genetic parameters (H^2^, GCV, PCV, GA and GAM%), rank the genotypes with MGIDI, model and interpret grain yield with a block-aware Gradient Boosting model, and validate the main biological yield drivers by comparing SHAP-based contributions with permutation importance.

## 2. Materials and Methods

### 2.1. Study Area

**Figure 1:**
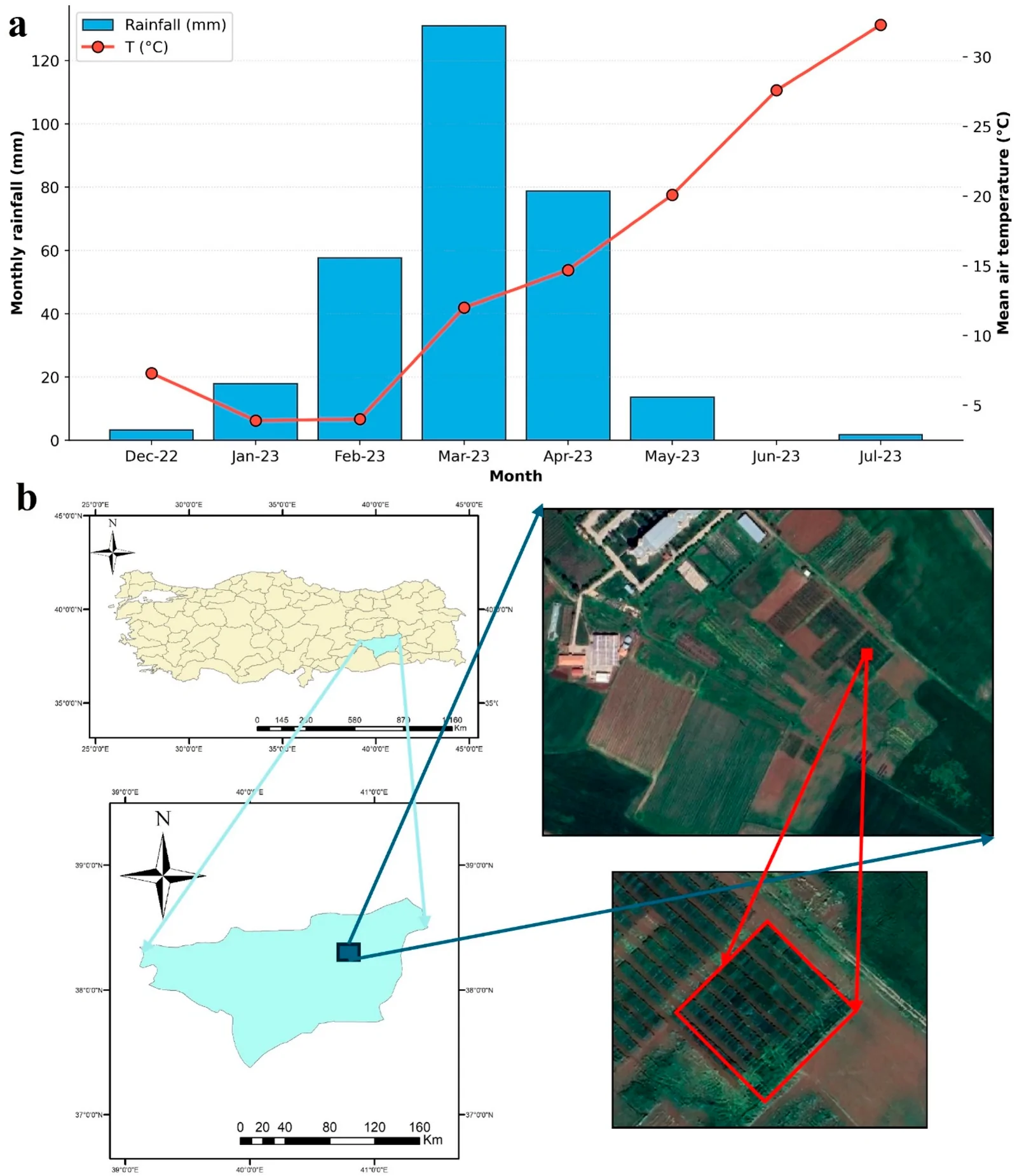
(**a**) Monthly rainfall and mean air temperature during the 2022–2023 growing season in Diyarbakır, Türkiye; (**b**) location of the study area.

The experiment was conducted during the 2022–2023 growing season at the experimental field of the Faculty of Agriculture, Dicle University (37°53′ N, 40°16′ E, 668 m a.s.l.) in Diyarbakır, Türkiye, which represents the semi-arid Mediterranean production conditions of Southeastern Anatolia. Total seasonal rainfall was 330.7 mm, of which 74% fell between December and April, whereas only 6.0 mm fell during the grain-filling period (June–July). Monthly rainfall and mean air temperature for the growing season, together with the location of the study area, are shown in Figure 1. In this study, “rainfed” refers to a production system in which crop water supply was derived exclusively from natural precipitation, with no supplementary irrigation applied at any stage of the crop cycle.

### 2.2. Plant Material

Twenty bread wheat (*Triticum aestivum* L.) genotypes were evaluated, comprising registered commercial cultivars, advanced breeding lines and local landraces (Table 1). The genotype set was chosen to represent the genetic diversity currently used in the region and to include candidates belonging to different phenological classes.

### 2.3. Soil Characteristics

Composite samples were taken from the 0–30 cm soil layer before sowing and analysed using standard physical and chemical methods. The experimental soil was slightly alkaline (pH 8.15), non-saline (EC 1.03 dS m^−1^), calcareous (CaCO_3_ 10.59%) and low in organic matter (0.77%). Total nitrogen, available phosphorus (Olsen method) and potassium were 0.04%, 6.00 mg kg^−1^ and 493.26 mg kg^−1^, respectively; DTPA-extractable micronutrients were Fe 8.86, Zn 0.29, Mn 23.10 and Cu 1.72 mg kg^−1^. The full set of soil properties is given in Table 2.

### 2.4. Experimental Design and Crop Management

The experiment was established in a randomised complete block design (RCBD) with four replications. Plots consisted of 6 rows × 4 m with 20 cm row spacing, and the sowing density was set at 500 seeds m^−2^. Sowing was carried out with a plot drill on 9 December 2022, and harvest was performed with a plot combine on 12 July 2023. Basal fertilisation was applied at sowing as 60 kg ha^−1^ N and 60 kg ha^−1^ P_2_O_5_ (20-20-0 compound), and 60 kg ha^−1^ N was top-dressed as urea (46% N) at stem elongation. Weed control was achieved with a mixture of 2,4-D ethylhexyl ester and florasulam. No irrigation was applied; the crop was grown entirely under rainfed conditions.

### 2.5. Trait Measurements

Days to heading (DH) were recorded as the number of days from sowing until spikes had fully emerged from the flag-leaf sheath in approximately 70% of the plants in a plot, and days to flowering (DF) were defined similarly, based on 70% anther extrusion; growth stages were assessed following the Zadoks decimal code [23]. The Normalised Difference Vegetation Index (NDVI) was measured at stem elongation (NDVI_SE) and at heading (NDVI_HD) with a GreenSeeker hand-held sensor (Trimble Inc., Sunnyvale, CA, USA) under clear-sky conditions between 11:00 and 15:00. Leaf area index (LAI) was determined at heading with a LAI-2200 Plant Canopy Analyzer (LI-COR Biosciences, Lincoln, NE, USA), taking two above-canopy and four below-canopy readings per plot. SPAD chlorophyll was measured at heading on the flag leaves of ten randomly selected plants per plot between 10:00 and 12:00 with a SPAD-502 metre (Konica Minolta, Tokyo, Japan). Plant height (PH) and spike length (SL) were measured at physiological maturity on ten randomly selected tillers per plot. The number of spikelets per spike (NSpS), number of grains per spike (NGS) and grain weight per spike (GWS) were determined on ten representative spikes per plot. Grain yield (GY, kg da^−1^) was obtained by mechanical harvest and expressed at 14% moisture, thousand-grain weight (TGW) was calculated from 1000 randomly selected grains per plot, and grain protein content was measured with a near-infrared transmittance (NIT) grain analyser (Infratec 1241; FOSS Analytical A/S, Hillerød, Denmark).

### 2.6. Statistical Analysis and Estimation of Genetic Parameters

Each trait was analysed with a mixed-effects model for the RCBD, y_ij_ = μ + B_i_ + G_j_ + ε_ij_, where B_i_ is the block effect and G_j_ is the genotype effect. Genotypic (σ^2^g) and phenotypic (σ^2^p) variances were estimated on an entry-mean basis from the genotype and error mean squares as σ^2^g = (MSg − MSe)/r and σ^2^p = MSg/r, where r = 4. Broad-sense heritability was calculated on an entry-mean basis as H^2^ = σ^2^g/σ^2^p, and genotypic and phenotypic coefficients of variation (GCV, PCV) were expressed as percentages of the grand mean. The expected genetic advance at 25% selection intensity was calculated as GA = *k* · σp · H^2^, where *k* = 1.271 is the standardised selection differential, and was also expressed as a percentage of the mean (GAM%). GAM% values were classified as low (<10%), moderate (10–20%) or high (>20%). Mean comparisons used the least significant difference (LSD, α = 0.05) test, and compact letter displays (CLD) based on the pairwise-comparison matrix were used to summarise homogeneous groups.

### 2.7. MGIDI Multi-Trait Selection

Trait values were rescaled to a 0–100 range in the direction of the ideotype. Grain yield, plant height, spike length, the number of spikelets per spike, the number of grains per spike, grain weight per spike, NDVI at stem elongation, NDVI at heading, LAI at heading, SPAD, thousand-grain weight and grain protein content were treated as “maximise” traits and rescaled as: rX = 100 × (X − Xmin)/(Xmax − Xmin). Days to heading and days to flowering were treated as “minimise” traits (earliness preferred to escape terminal drought) and rescaled as: rX = 100 × (Xmax − X)/(Xmax − Xmin). In both cases, rescaled values of 100 correspond to the ideotype and values of 0 to the least desirable genotype. Before rescaling, all traits were averaged over replications to obtain genotype means, and the rescaled dataset was standardised. Varimax-rotated factor analysis was applied to the standardised data, retaining factors with eigenvalues greater than 1; latent factors were extracted by principal component analysis and rotated orthogonally (varimax) to improve interpretability. For each genotype, the MGIDI was computed as MGIDIi = √(Σ(Fij − Fj)^2^), where F is the ideotype vector in the rotated factor space, defined as a hypothetical genotype with the maximum (100) value for all rescaled traits. Factor contributions to MGIDI were computed as the proportional share of each factor in the squared Euclidean distance. The five genotypes with the lowest MGIDI values, corresponding to a selection intensity of 25%, were considered selected. The selection differential (SD) and selection gain (SG%) were calculated according to Equations (1) and (2), respectively.

(1) $$SD={\bar{x}}_{sel}-\bar{x}pop$$

(2) $$SG\%=100\times \frac{SD}{\bar{x}pop}$$

Because the analysis was carried out on 20 genotypes and 14 traits (an observation-to-variable ratio of about 1.4:1), the factor solution should be regarded as descriptive of this particular dataset rather than as a stable estimate of a broader latent structure, and this constraint is acknowledged in the Section 4.5.

### 2.8. Machine Learning Model for Grain Yield

Grain yield was used as the response variable and the other 13 traits as explanatory variables, giving a dataset of 80 plot-level observations (20 genotypes × 4 blocks). Outliers were examined with the 3 × IQR rule, and none were removed; no high-leverage points were detected (Cook’s distance < 4/n). Missing values were imputed with the median, and all variables were standardised within a scikit-learn Pipeline. Three tree-based learners (XGBoost, Gradient Boosting, Random Forest) and two regularised linear models (Ridge, ElasticNet) were evaluated. Because the RCBD induces intra-block spatial correlation, a random-fold cross-validation would allow observations from the same block to appear in both training and test sets and would therefore over-estimate the true predictive skill; a block-stratified GroupKFold scheme was adopted in which each replication block was used as one test fold, so that the folds mirror the spatial structure of the field trial and prevent this form of data leakage. Performance was quantified by R^2^, RMSE and MAE, and cross-validated predictions were obtained with cross_val_predict. Hyper-parameters of the tree-based learners were tuned by manual grid search over 12 regularisation-focused candidate configurations for each algorithm; the search spaces were: number of estimators {100, 150, 200, 300}, maximum depth {2, 3, 4}, learning rate {0.02, 0.03, 0.05, 0.08}, subsample fraction {0.6, 0.7, 0.8, 0.9}, column-subsample fraction {0.6, 0.7, 0.8, 0.9} and the algorithm-specific regularisation terms (e.g., min_child_weight {2, 3, 5, 7}, gamma {0, 0.05, 0.1, 0.2}, reg_α {0.1, 0.5, 1.0, 2.0}, reg_λ {1.0, 2.0, 3.0, 5.0} for XGBoost; comparable ranges for Gradient Boosting and Random Forest). For Ridge and ElasticNet the regularisation strength α was scanned over {0.1, 0.5, 1.0, 2.0, 5.0} and, for ElasticNet, l1_ratio over {0.2, 0.5, 0.8}. The configuration with the highest block-stratified CV R^2^ was retained. The best-performing tree-based model in cross-validation (Gradient Boosting) was selected as the final model and interpreted with both permutation importance (30 repeats, random_state = 42) and TreeSHAP; SHAP values were summarised as mean absolute values, and beeswarm and dependence plots were used to analyse the magnitude and direction of each variable’s contribution. To ensure reproducibility, all analyses used a fixed random seed (random_state = 42) for cross-validation splitting, hyper-parameter tuning and permutation importance. Analyses were performed in Python 3.11 with pandas 2.2, NumPy 1.26, scikit-learn 1.5, xgboost 3.0, shap 0.45, statsmodels 0.14, seaborn 0.13 and matplotlib 3.10.

## 3. Results

### 3.1. Analysis of Variance and Genetic Parameter Estimates

Grain yield averaged 444.50 kg da^−1^, plant height 103.19 cm, and spike length 9.33 cm. The error coefficient of variation (CV%) ranged from 1.10% to 11.10%, with the lowest value for days to heading (1.10%) and the highest for LAI at heading (11.10%). Broad-sense heritability (H^2^) was high for all traits, ranging from 0.84 to 0.99 (Table 3). Genotypic coefficients of variation (GCV) ranged from 2.13% to 17.67% and phenotypic coefficients of variation (PCV) ranged from 2.20% to 18.22%, with the two parameters close to each other for each trait. For genetic advance (GA), the highest value was recorded for grain yield (76.60) and the lowest for NDVI at stem elongation (0.03). Genetic advance as a percentage of the mean (GAM%) ranged from 2.62% to 21.79%, with the highest value for grain weight per spike (21.79%), followed by the number of grains per spike (20.67%) and grain yield (17.23%). According to the GAM% classification, the number of grains per spike and grain weight per spike fell into the high class; grain yield, spike length, LAI and thousand-grain weight into the moderate class; and the remaining traits into the low class (Table 3).

### 3.2. Phenological, Physiological, Morphological and Yield-Related Traits

**Figure 2:**
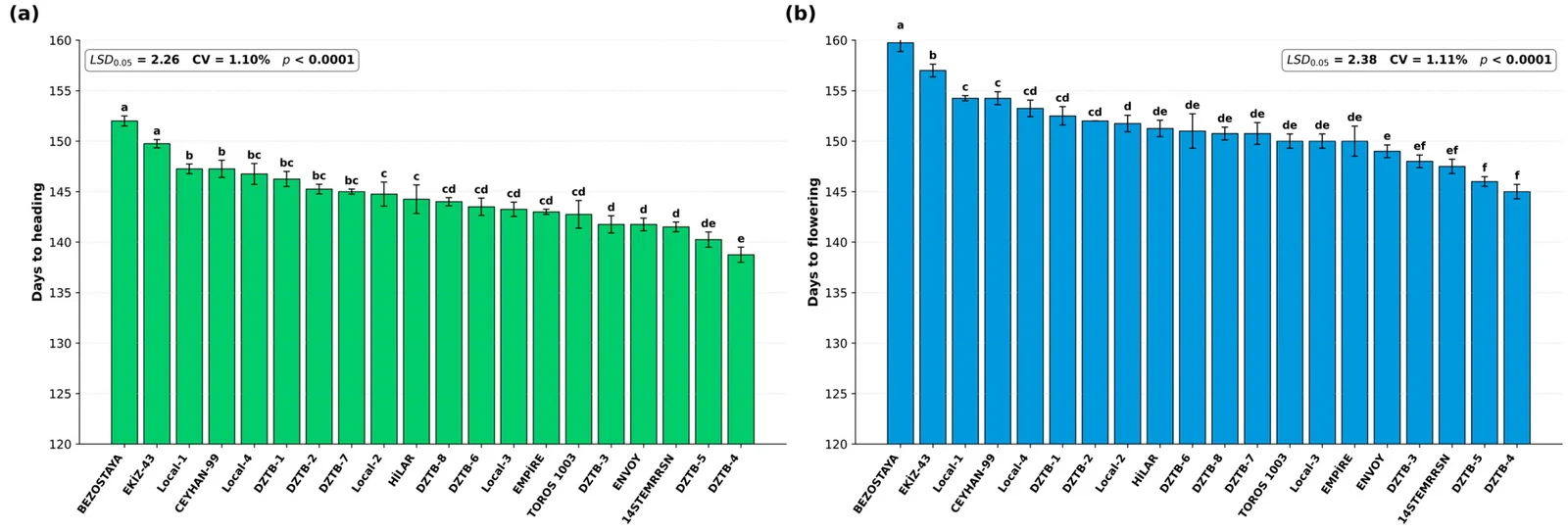
Phenological traits of the wheat genotypes: (**a**) days to heading; (**b**) days to flowering. Bars represent means ± SEM; different lowercase letters above bars indicate significant differences among genotypes according to Fisher’s LSD test at *p* < 0.05. LSD_0.05_, coefficient of variation (CV%) and *p*-value from one-way ANOVA are shown in the top-left inset of each panel.

Days to heading differed significantly among genotypes (LSD_0.05_ = 2.26; range 139–152 d), with the earliest heading in DZTB-4 and the latest in BEZOSTAYA; days to flowering followed the same ranking (LSD_0.05_ = 2.38; range 145–160 d). The two phenological traits were strongly and positively coupled, so early-heading genotypes also flowered early (Figure 2). Full genotype means with letter separations are given in Supplementary Table S1.

**Figure 3:**
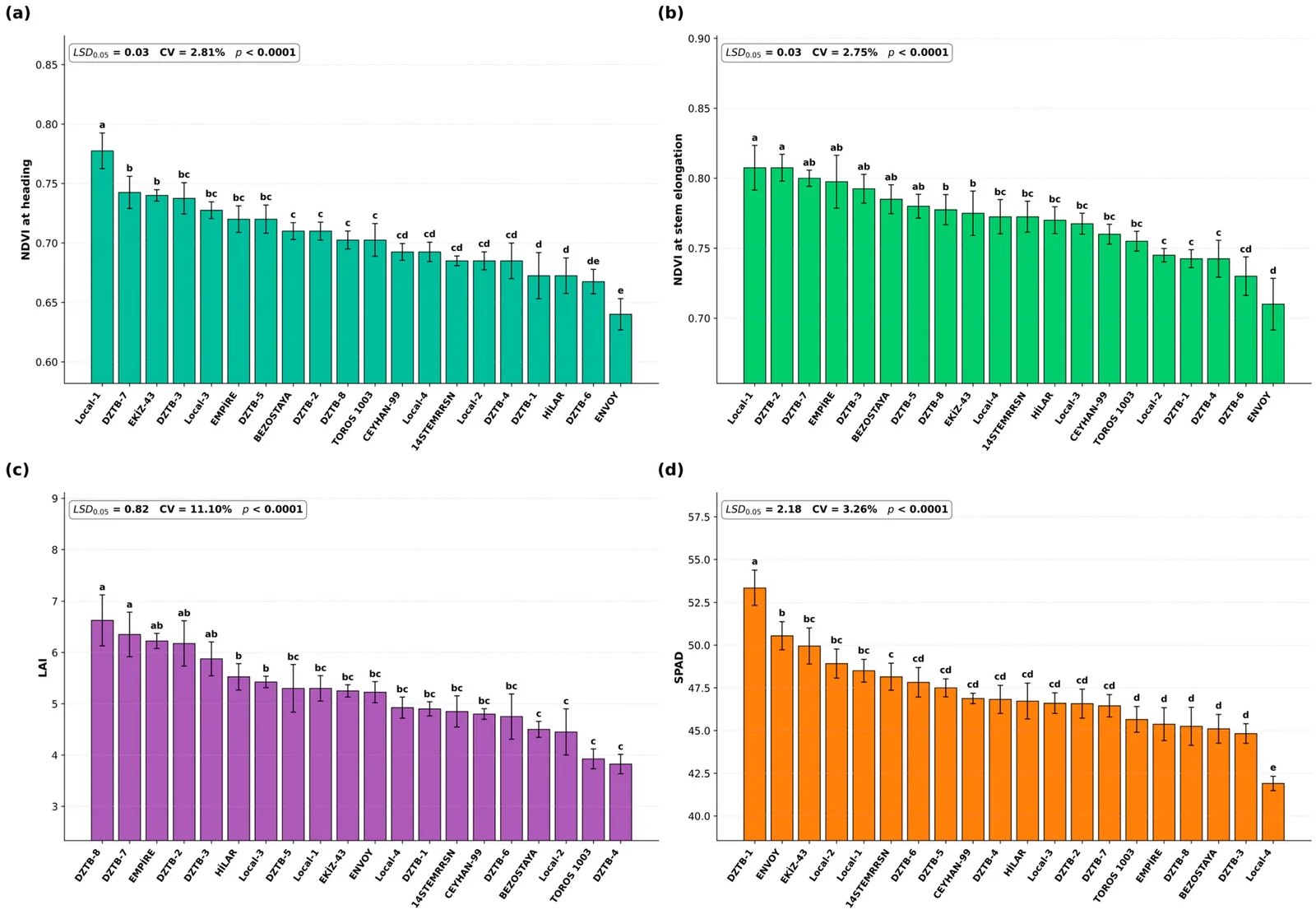
Sensor-based physiological traits: (**a**) NDVI at heading; (**b**) NDVI at stem elongation; (**c**) leaf area index (LAI) at heading; (**d**) SPAD chlorophyll at heading. Bars represent means ± SEM; different lowercase letters above bars indicate significant differences among genotypes according to Fisher’s LSD test at *p* < 0.05. LSD_0.05_, coefficient of variation (CV%) and *p*-value from one-way ANOVA are shown in the inset of each panel.

Genotypes differed significantly for all sensor-based physiological traits (Figure 3): NDVI at heading (0.64–0.78), NDVI at stem elongation (0.71–0.81), LAI (3.8–6.6) and SPAD (42–53). Local-1 recorded the highest NDVI values and ENVOY the lowest, while LAI and SPAD showed contrasting genotype rankings, consistent with the two indices capturing partially different components of canopy performance. Genotype-level means are provided in Supplementary Table S1.

**Figure 4:**
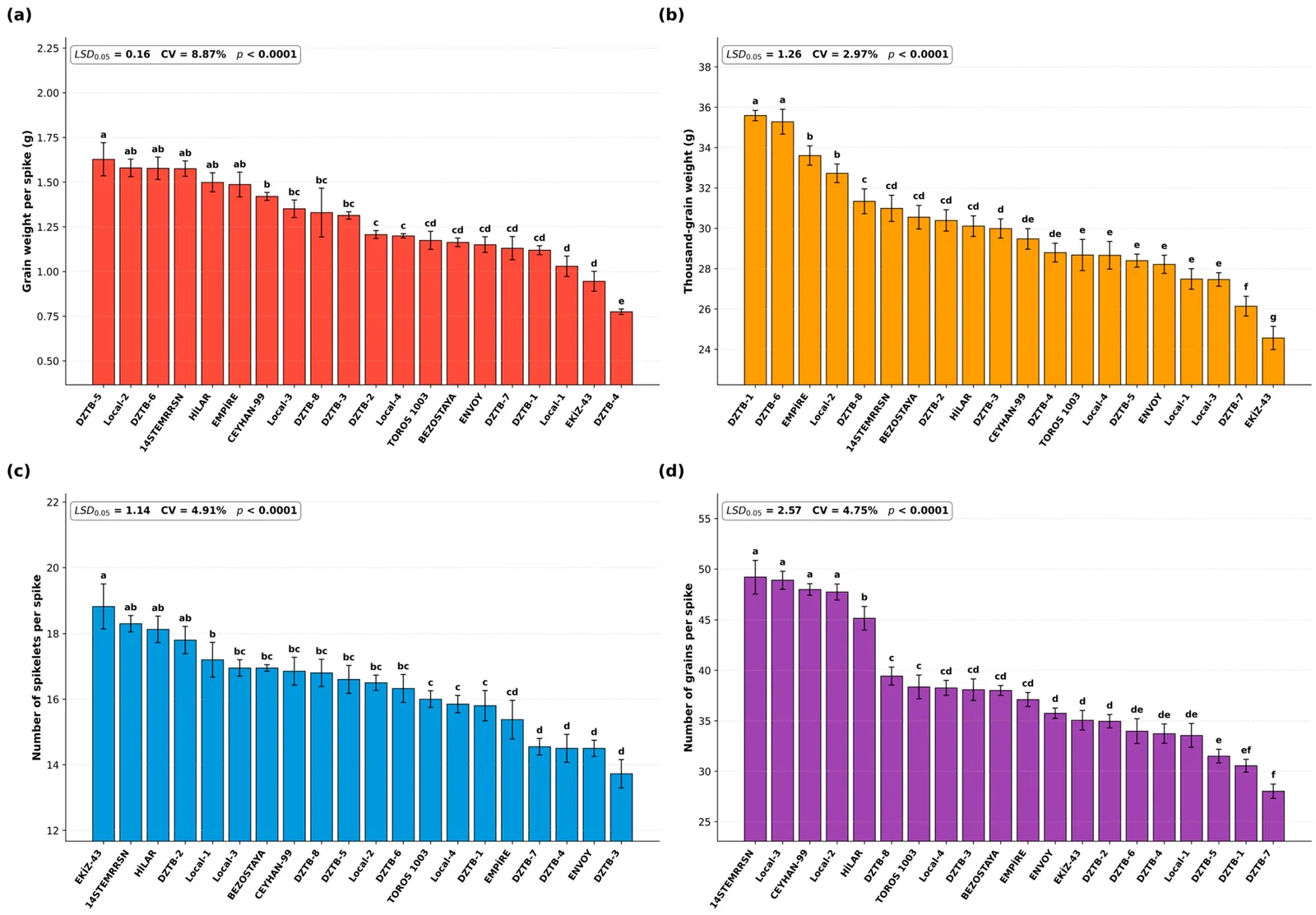
Yield components: (**a**) grain weight per spike; (**b**) thousand-grain weight; (**c**) number of spikelets per spike; (**d**) number of grains per spike. Bars represent means ± SEM; different lowercase letters above bars indicate significant differences among genotypes according to Fisher’s LSD test at *p* < 0.05. LSD_0.05_, coefficient of variation (CV%) and *p*-value from one-way ANOVA are shown in the inset of each panel.

Grain weight per spike (0.75–1.65 g) and thousand-grain weight (24.5–35.8 g) were the yield components with the widest genotypic range (Figure 4). DZTB-5 combined the highest grain weight per spike, DZTB-7 the highest thousand-grain weight, EKİZ-43 the highest number of spikelets per spike, and 14STEMRRSN the highest number of grains per spike (28–49 across the panel), pointing to distinct paths of yield formation among the leading genotypes.

**Figure 5:**
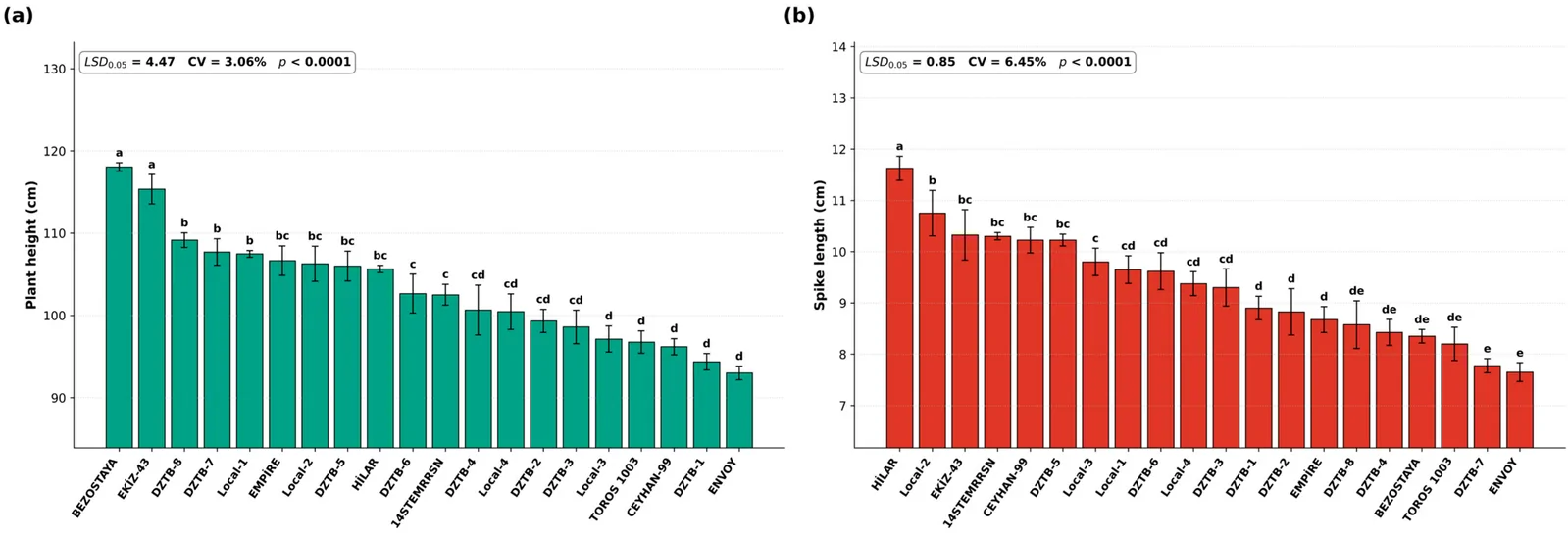
Plant morphology: (**a**) plant height; (**b**) spike length. Bars represent means ± SEM; different lowercase letters above bars indicate significant differences among genotypes according to Fisher’s LSD test at *p* < 0.05. LSD_0.05_, coefficient of variation (CV%) and *p*-value from one-way ANOVA are shown in the inset of each panel.

Plant height ranged from 93 cm (ENVOY) to 118 cm (BEZOSTAYA) and spike length from 7.6 cm (ENVOY) to 11.6 cm (HİLAR), with genotype effects significant at α = 0.05 in both cases (Figure 5).

**Figure 6:**
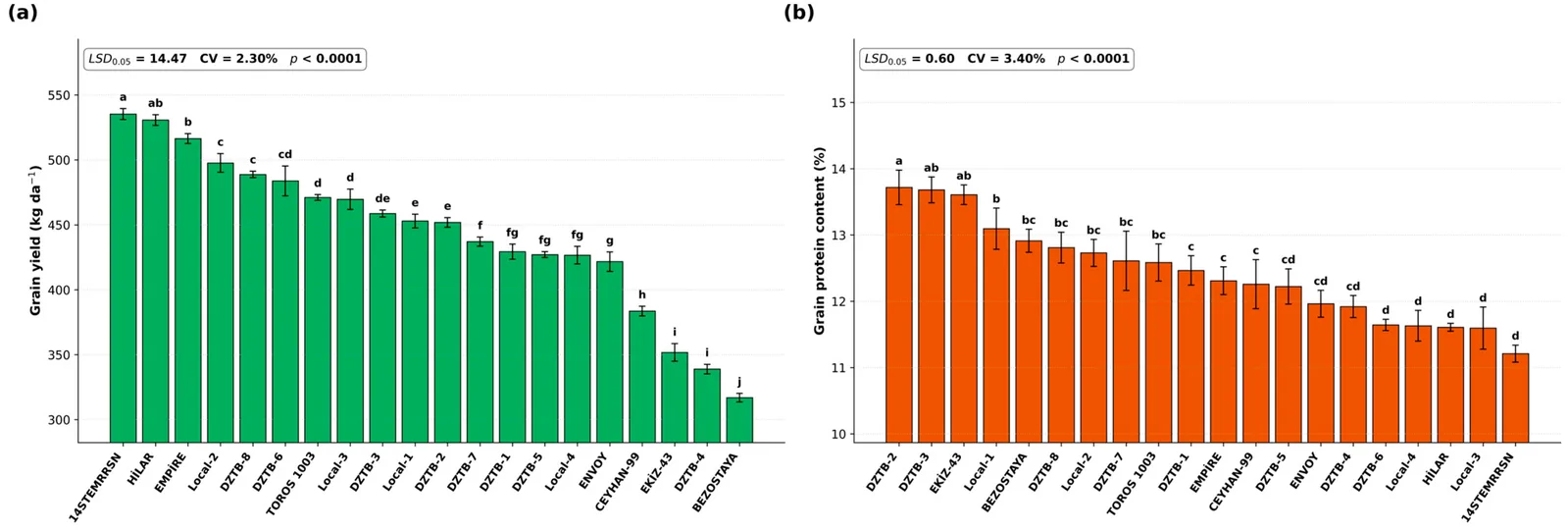
Grain yield and quality: (**a**) grain yield; (**b**) grain protein content. Bars represent means ± SEM; different lowercase letters above bars indicate significant differences among genotypes according to Fisher’s LSD test at *p* < 0.05. LSD_0.05_, coefficient of variation (CV%) and *p*-value from one-way ANOVA are shown in the inset of each panel.

Grain yield ranged from 320 kg da^−1^ (BEZOSTAYA) to 535 kg da^−1^ (14STEMRRSN), i.e., a ~1.7-fold spread across the panel, whereas grain protein content varied inversely (11.2–13.8%), with DZTB-2 and DZTB-3 having the highest and 14STEMRRSN the lowest (Figure 6). This pattern is consistent with the widely reported negative association between grain yield and protein concentration in bread wheat and motivated a formal quantitative assessment of the trade-off, reported in Section 3.4.

### 3.3. Multivariate Structure and Trait Associations

**Figure 7:**
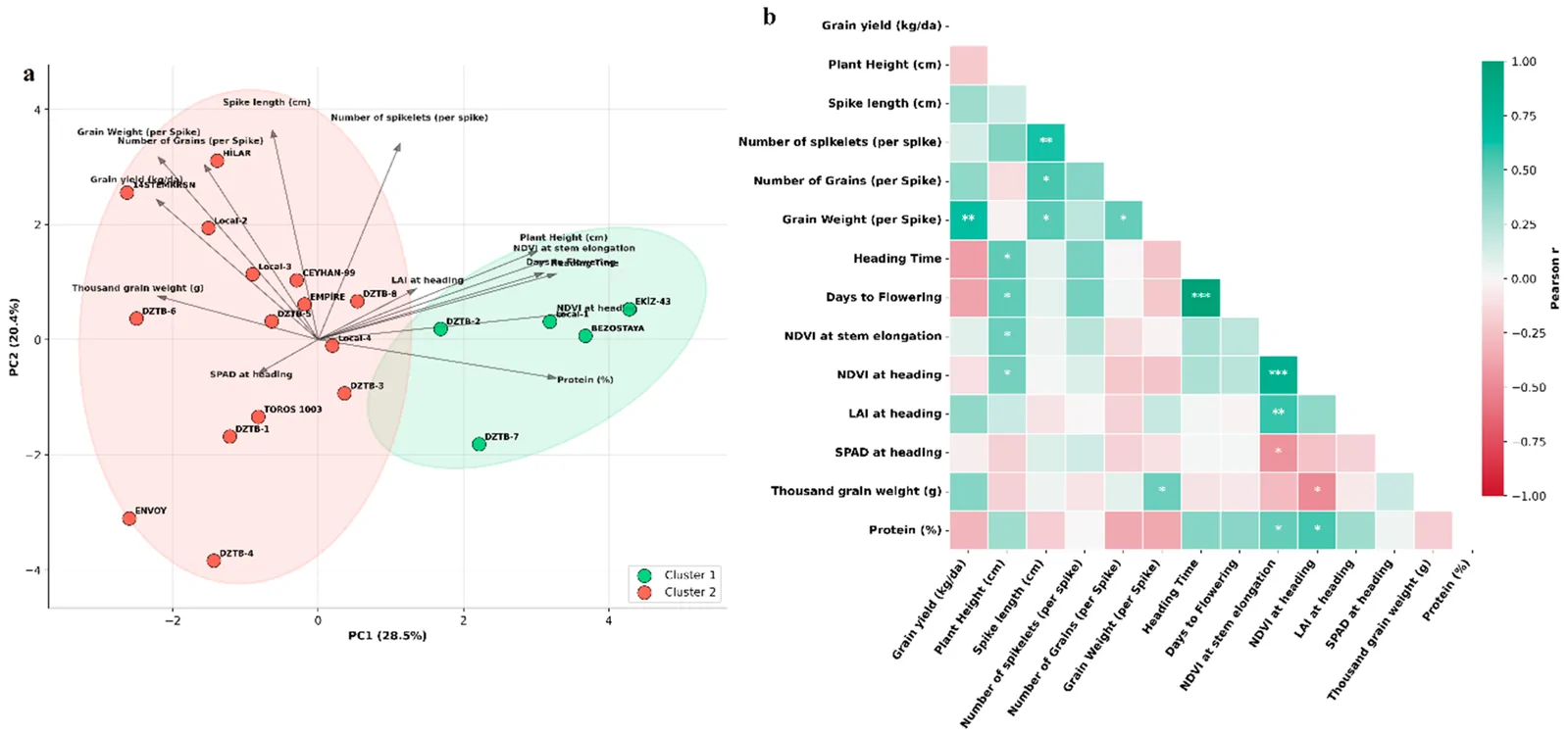
(**a**) PCA biplot of wheat genotypes and traits; (**b**) Pearson correlation matrix; asterisks denote * *p* < 0.05, ** *p* < 0.01, *** *p* < 0.001.

The first two principal components explained 48.9% of the total variance (PC1 = 28.5%, PC2 = 20.4%). In the PCA biplot, genotypes clustered into two main groups: one associated more with plant height, late heading and high protein content, and the other with higher yield and yield-component traits (Figure 7a). Grain yield was positively associated with grain weight per spike, and the number of spikelets per spike was positively associated with the number of grains per spike. A strong positive correlation was found between NDVI at stem elongation and NDVI at heading, and a high positive association was observed between days to heading and days to flowering. Grain protein content was negatively associated with grain yield (Figure 7b).

### 3.4. MGIDI-Based Multi-Trait Selection

Five factors explaining 85.9% of the total variance were retained, accounting for 21.3%, 21.3%, 19.0%, 14.8% and 9.5% of the variance, respectively (Table 4). The first factor (FA1) showed high negative loadings for days to heading (−0.980) and days to flowering (−0.990), with a positive loading for plant height (0.599). The second factor (FA2) showed high positive loadings for NDVI at stem elongation (0.899), NDVI at heading (0.822) and LAI (0.807), as well as grain protein content (0.610). The third factor (FA3) had high loadings for spike length (0.921), the number of spikelets per spike (0.787) and the number of grains per spike (0.753). The fourth factor (FA4) was associated with grain yield (0.653), grain weight per spike (0.696) and thousand-grain weight (0.894), and the fifth factor (FA5) was represented by SPAD (0.975) (Table 4).

**Figure 8:**
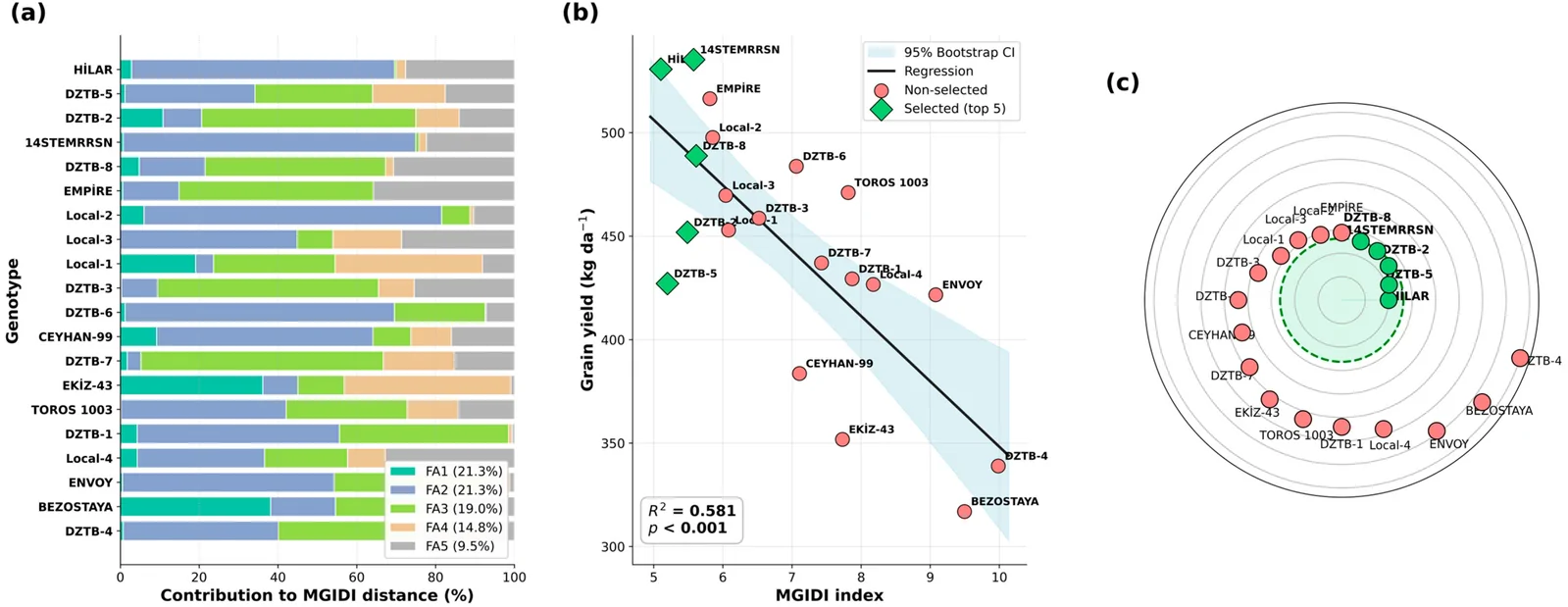
MGIDI analysis. (**a**) Proportional contribution of the five retained factors to the MGIDI distance for each genotype; (**b**) Relationship between MGIDI and grain yield; (**c**) Polar plot of the MGIDI ranking; the cut-off separates selected from non-selected genotypes.

The proportional factor contributions to MGIDI differed among genotypes (Figure 8a). The relationship between MGIDI and grain yield was negative and statistically significant (Figure 8b). The genotype ranking based on MGIDI is shown on the polar plot, with genotypes below the defined threshold separated as selected; the genotypes with the lowest MGIDI values were HİLAR, DZTB-5, DZTB-2, 14STEMRRSN and DZTB-8 (Figure 8c).

In the selected genotypes, mean grain yield was 486.75 kg da^−1^, corresponding to a 42.24 kg da^−1^ increase over the population mean and a selection gain of 9.50%. Grain weight per spike, LAI, spike length and the number of spikelets per spike increased by 12.88%, 9.31%, 6.23% and 7.01%, respectively; the number of grains per spike increased by 4.66% and plant height by 1.29%. Days to heading and days to flowering decreased by 1.40 and 1.70 days, corresponding to changes of 0.97% and 1.12% in the desired (earliness) direction. NDVI at stem elongation and thousand-grain weight increased by 1.56% and 1.08%, respectively, whereas NDVI at heading, SPAD and grain protein content decreased by 0.87%, 0.65% and 0.93% (Table 5).

Although the number of grains per spike showed a high genetic advance (GAM% = 20.67), its realised selection gain in the MGIDI-selected set was only +4.66% (Table 5). This apparent discrepancy reflects the structure of the factor solution rather than a weakness of MGIDI: the number of grains per spike loaded predominantly on the spike-architecture factor (FA3), which co-varied moderately with the yield-sink factor (FA4) but weakly with the canopy factor (FA2). Because MGIDI ranks genotypes on their joint distance to the ideotype across all five factors, a trait whose genotypic variation is largely aligned with one factor can be “outvoted” by other traits that better balance the remaining factors. This is a general property of ideotype-based indices: high theoretical GAM% (which reflects the univariate genetic potential of a trait) does not necessarily translate into a large realised gain (which reflects the multivariate covariance structure), a distinction breeders should keep in mind when combining direct and multi-trait selection.

Within the MGIDI-selected set, the yield–protein trade-off was explicitly quantified. Selected genotypes recorded a mean grain yield of 486.8 kg da^−1^ against 444.5 kg da^−1^ for the whole population (+9.50%), while mean grain protein declined only from 12.43% to 12.31% (SG% = −0.93%). The Pearson correlation between grain yield and grain protein across the 20 genotypes was moderate and negative (r = −0.31, *p* = 0.19), and the linear regression of protein on yield gave a slope of −0.0053% per kg da^−1^ (95% CI: −0.014 to +0.004). In practical terms, the 42 kg da^−1^ yield gain delivered by MGIDI is expected to reduce grain protein by less than 0.25 percentage points, which is markedly milder than the trade-off typically reported for yield-only selection in bread wheat and provides direct evidence that the multi-trait ideotype framework preserves grain quality while advancing productivity.

### 3.5. Machine Learning Prediction of Grain Yield

Among the models, the highest accuracy was obtained with Gradient Boosting (CV R^2^ = 0.740). XGBoost, Random Forest, ElasticNet and Ridge achieved CV R^2^ values of 0.703, 0.658, 0.546 and 0.526, respectively (Table 6). On the same data, the training R^2^ of the Gradient Boosting model was 0.998, yielding a training-versus-cross-validation R^2^ gap of 0.26. This gap is noticeable but not unusual for a tree-ensemble model fitted to 80 observations and 13 explanatory variables, and it was substantially smaller than the >0.28 gap observed under looser regularisation. The gap is interpreted transparently in the Discussion as an unavoidable consequence of the modest sample size rather than as evidence that the cross-validated skill is spurious.

**Figure 9:**
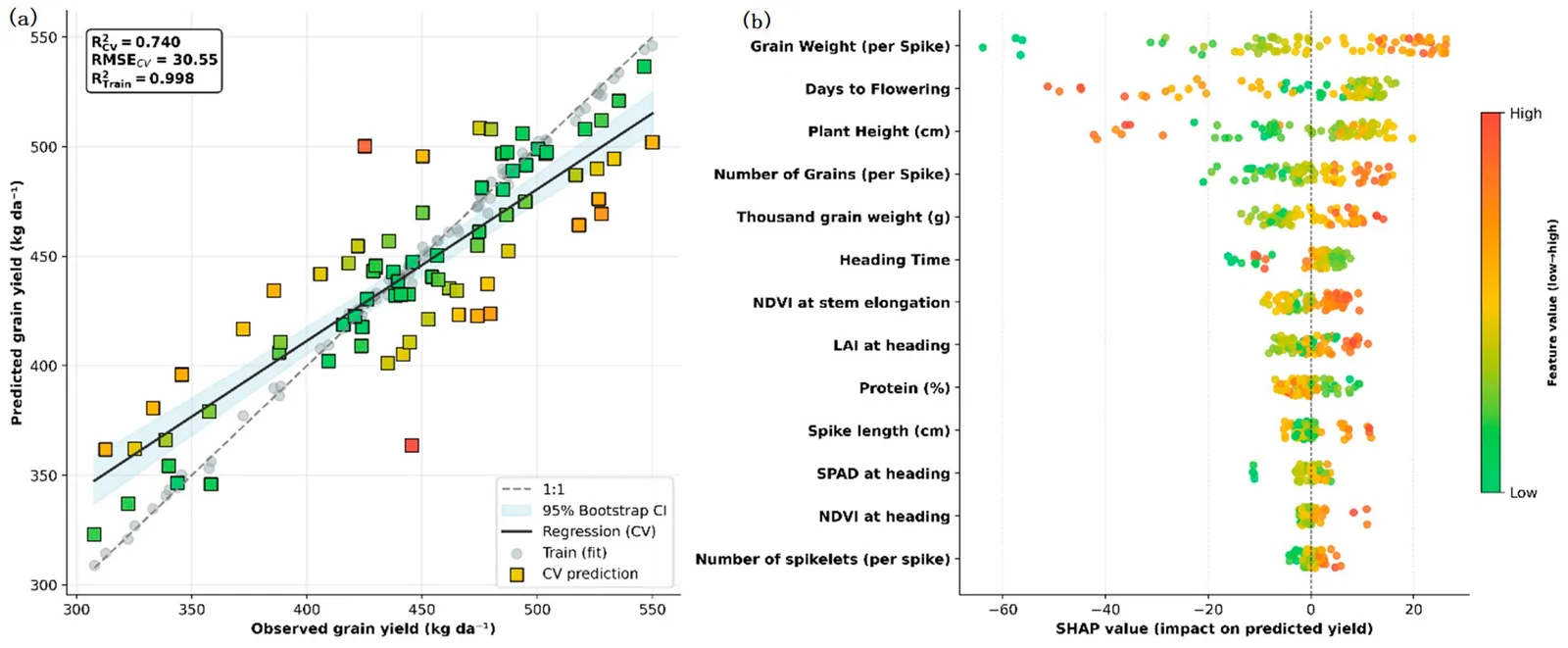
Explainable machine learning prediction of grain yield. (**a**) Observed vs. predicted grain yield for the Gradient Boosting model; (**b**) SHAP beeswarm summary plot.

When permutation importance and SHAP values were considered together, the most important variable was grain weight per spike, followed by plant height and days to flowering. Days to heading, thousand-grain weight, and the number of grains per spike were of intermediate importance, whereas traits such as LAI, NDVI, and protein made smaller contributions (Table 7). The Gradient Boosting model achieved an RMSE of 30.55 kg da^−1^ and an R^2^ of 0.740 (Figure 9a), and the distribution of the effects of the variables on predicted grain yield is presented in the SHAP summary plot (Figure 9b).

### 3.6. Genotype × Trait Clustering

**Figure 10:**
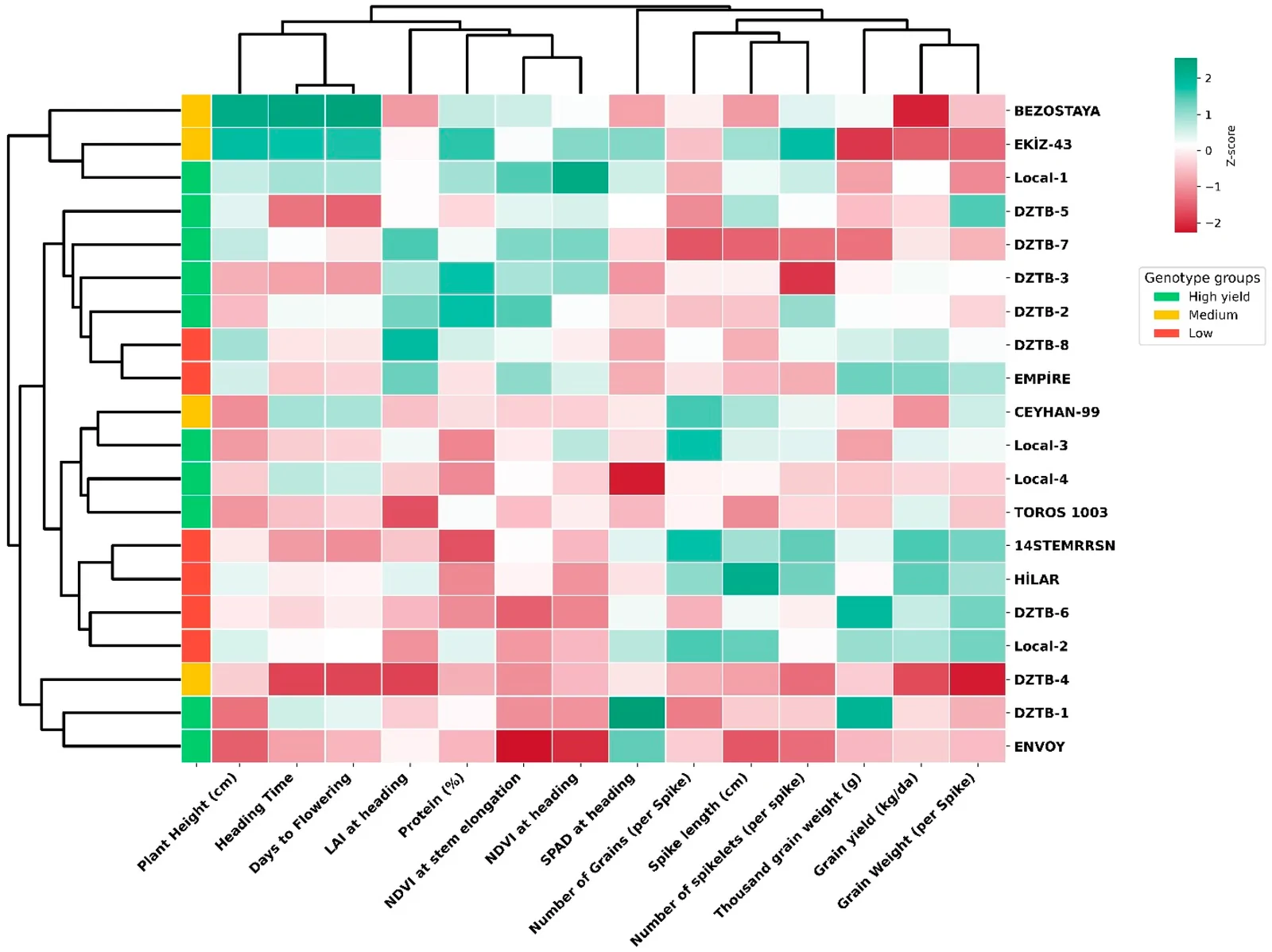
Hierarchical clustering heatmap of wheat genotypes and traits based on Z-scored means (Euclidean distance, average linkage).

Genotypes were grouped into three main clusters according to their similarity: the high-, medium- and low-yielding groups. The high-yielding group comprised 14STEMRRSN, HİLAR, EMPİRE, Local-2, DZTB-8 and DZTB-6, whereas the low-yielding group comprised BEZOSTAYA, DZTB-4 and EKİZ-43. Traits were grouped into three main clusters: phenological traits and plant height in the first; NDVI, LAI and protein in the second; and yield components in the third. The Z-score-based heatmap showed that high-yielding genotypes had high values for yield and yield components, whereas low-yielding genotypes had lower values for these traits (Figure 10).

## 4. Discussion

### 4.1. Genotypic Variation, Heritability and Genetic Gain

The high broad-sense heritability (H^2^ = 0.84–0.99) and the close agreement between genotypic and phenotypic coefficients of variation indicate that the traits examined were largely controlled by genetic factors, with limited environmental influence. The low error coefficients of variation and the structure of the experimental design also point to reliable phenotypic measurements and to genotypic differences having been captured accurately. It should nonetheless be kept in mind that these estimates are entry-mean heritabilities obtained in a single environment; because the genotype × environment component cannot be separated from the genotypic component in such a design, they represent upper bounds rather than parameters transferable to other environments. Consistent with the general pattern, high heritability in wheat has been reported to increase the efficiency of selection [24].

The similarity between GCV and PCV suggests that phenotypic variation was largely due to genetic variation and that selection accuracy was high, supporting effective selection for yield and yield components [17]. The combination of high heritability with moderate-to-high GCV for grain yield, the number of grains per spike, and grain weight per spike indicates that meaningful genetic gain can be achieved for these traits, and their high GAM% values suggest that yield improvement may be achieved more effectively through yield components than through direct selection on yield, supporting multi-trait selection as a useful strategy [22]. Despite high heritability, the low GCV and GAM% values for phenological traits indicate limited genetic variation in these traits; nonetheless, under semi-arid conditions, early heading and flowering represent an important adaptation mechanism for escaping terminal drought [25]. The significant differences among genotypes for all traits indicate substantial genetic variation that can be exploited in breeding programmes, consistent with reports of considerable variation among wheat genotypes under drought conditions [10].

### 4.2. MGIDI as a Balanced Multi-Trait Selection Tool

The 9.50% increase in grain yield in the MGIDI-selected genotypes, together with simultaneous improvements in spike architecture and physiological traits, indicates a balanced genetic gain rather than the trade-offs typical of single-trait selection. This suggests an advantage of MGIDI over classical selection approaches, in which yield gains often come at the expense of other traits. The magnitude of this gain aligns with values recently reported for MGIDI-based selection in wheat under rainfed and moisture-limited conditions: Al-Ashkar et al. [13] reported MGIDI-driven yield increments of 5.5–8.4% across drought-stressed environments, Farhad et al. [26] documented a 6.9% gain in an early-sown ideotype trial, and Meier et al. [24] reported 8–10% gains under variable rainfall conditions. The current 9.50% increase therefore sits at the upper end of this benchmark range for rainfed wheat. Notably, in the present study, this gain was accompanied by coordinated gains across grain weight per spike (12.88%), LAI (9.31%), spikelets per spike (7.01%) and spike length (6.23%), and by a protein loss of less than 1% a pattern rarely achieved by single-trait yield selection, which typically trades gains in one trait for losses in others. The factor-analytic backbone of MGIDI is a plausible mechanism for this behaviour: because the index balances distance to the ideotype across orthogonal (varimax-rotated) factors that respectively capture phenology, canopy activity, spike architecture and sink size, extreme values in a single trait cannot dominate the ranking, and genotypes with balanced, multi-trait performance are systematically preferred.

The factor-analytic basis of MGIDI reduces multicollinearity among traits and ranks genotypes according to their Euclidean distance to the ideotype in the rotated factor space, clearly revealing each genotype’s strengths and weaknesses. Studies in various crops likewise indicate that MGIDI is an effective and practical decision-support tool for evaluating multiple traits jointly [22,26]. In this way, MGIDI not only ranks genotypes but can also support breeders in developing targeted selection strategies. The factor solution should nonetheless be interpreted with care: the analysis was carried out on 20 genotypes and 14 traits (observation-to-variable ratio ≈ 1.4:1), which is below the ratios typically recommended for stable factor extraction. Although eigenvalue-based factor retention and varimax rotation partly mitigate this constraint, replicated analyses in multi-environment trials with larger germplasm panels will be needed to confirm the stability of the five-factor structure and of the associated trait–factor assignments.

The significant negative relationship between MGIDI and grain yield (R^2^ = 0.58) indicates that, despite balancing multiple traits, the index retained appreciable discriminatory power for yield. Three of the five MGIDI-selected genotypes (14STEMRRSN, HİLAR and DZTB-8) also belonged to the high-yielding cluster identified in the clustering analysis, and the selected group as a whole yielded above the population mean; the index therefore recovered part, but not all, of the highest-yielding material, which is the expected behaviour of an index that balances yield against spike architecture, phenology and canopy traits. This partial rather than complete overlap is particularly relevant in wheat, where a negative relationship between yield and quality is common [27]. At the same time, MGIDI results should be interpreted with the environment in mind: because this study was conducted at a single location in a single year, the values obtained are specific to this environment, and evaluation in multi-environment trials together with stability indices would provide more reliable selection decisions.

### 4.3. Explainable Machine Learning Identifies the Biological Drivers of Yield

The Gradient Boosting model provided moderate-to-high accuracy in predicting grain yield (R^2^ ≈ 0.74), illustrating the usefulness of tree-based algorithms for capturing non-linear relationships. Given that yield arises from complex interactions among phenological, physiological and environmental factors, such models are expected to perform better than classical linear approaches [28], and studies using multi-source datasets have similarly reported high accuracy and generalisability for machine learning yield prediction [29]. The main contribution here, however, lies not only in predictive performance but also in interpretability: SHAP analysis quantified how each variable shaped the model output, enabling the interpretation of the mechanisms underlying yield formation. Explainable AI approaches are reported to be useful for understanding complex relationships in agricultural data and to add value to decision-support systems [30].

According to the SHAP results, the most important variables determining grain yield were grain weight per spike, plant height and days to flowering, consistent with yield formation being closely linked to source–sink relationships and photosynthetic capacity. The decisive role of yield components, particularly grain weight and grain number, has been emphasised in many studies [5], and the effects of plant height and phenological traits relate to adaptation and growth duration, which are important determinants under semi-arid conditions [10]. The lower but non-negligible contributions of physiological indicators such as NDVI, LAI and SPAD suggest that these variables contribute indirectly to yield formation, reflecting plant growth and photosynthetic activity and thus playing a complementary role [21]. The convergence of SHAP-based rankings with permutation-importance results indicates that the yield drivers identified by the model are consistent and biologically meaningful, in line with reports that explainable machine learning offers advantages in both predictive power and interpretability [31]. It should nevertheless be emphasised that the model was fitted to 80 plot-level observations from a single environment; the cross-validated R^2^ of 0.74 therefore describes the model’s ability to generalise across blocks within this trial and not across seasons or locations, and yield prediction is known to be strongly influenced by spatial and temporal variability [32]. The gap between the training R^2^ (0.998) and the cross-validated R^2^ (0.740) 0.26 in absolute terms deserves an explicit comment. Tree-boosting models are known to interpolate training observations closely when the sample size is small, and the training R^2^ should therefore not be interpreted as an estimate of out-of-sample skill; the block-stratified CV R^2^ of 0.74 is the relevant metric for generalisation across blocks within the trial. The reported manual-tuning grid (Section 2.8) was deliberately biassed toward stronger regularisation (learning_rate ≤ 0.08; max_depth ≤ 4; large reg_α and reg_λ; large min_child_weight) to keep the CV–train gap as small as possible without sacrificing predictive accuracy, and the reported model corresponds to the smallest gap achievable in this search space. A larger sample from multi-year and multi-location trials would be the most direct means of narrowing this gap further and confirming the generalisability of the SHAP-based trait ranking.

### 4.4. Integrating MGIDI with Explainable Machine Learning: Implications for Breeding

This study presents an integrated framework in which MGIDI and SHAP-based explainable machine learning are used together under rainfed wheat conditions. The two approaches are complementary: MGIDI enables selection based on multiple traits, while explainable machine learning reveals the key traits determining yield formation. The prominence of spike-architecture traits in the MGIDI results and the identification of grain weight per spike as the dominant variable in the SHAP analysis indicate that the selection decisions are biologically consistent.

Evaluating MGIDI and machine learning outputs together links genotype selection with trait-based breeding strategies and can simplify selection when many traits must be considered, jointly, by focusing on a limited number of high-impact traits. Multi-trait selection approaches have similarly been reported to be effective in improving complex traits such as yield and quality together [27]. Overall, integrating MGIDI with explainable machine learning supports not only the identification of superior genotypes, but also an understanding of the biological mechanisms underlying that superiority, and can thereby strengthen decision-support processes in wheat breeding programmes. Two of the five MGIDI-selected genotypes HİLAR and 14STEMRRSN combine high multi-trait scores with above-mean grain yield and are therefore of immediate practical interest for breeding programmes in the Southeastern Anatolia rainfed region. HİLAR is already a registered Turkish cultivar with a proven adaptation record; its top MGIDI ranking supports its continued deployment as a commercial reference cultivar and as a recurrent parent for future crosses aimed at combining spike architecture with earliness. 14STEMRRSN, a CIMMYT-derived advanced line selected in a stem rust resistance nursery, is a strong candidate for further evaluation as a parental line, particularly for pyramiding high grain weight per spike and moderate phenology into locally adapted backgrounds. DZTB-2, DZTB-5 and DZTB-8, all advanced breeding lines from Dicle University, warrant priority testing in multi-environment trials to assess yield stability under contrasting rainfall regimes. Formal registration or commercial release recommendations will, however, require multi-year, multi-location confirmation of these outcomes.

### 4.5. Limitations

These results should be considered within the study’s limitations. First, because the study relied on data from a single location and a single year, the genotype × environment interaction (G × E) could not be analysed, and genotype performance may differ across environments; supporting these results with multi-environment trials would lead to more reliable selection decisions. Second, the limited number of observations may constrain the generalisation capacity of the machine learning models, and studies with multi-year and larger datasets would allow the results to be validated more robustly [29]. Third, all explanatory variables in the model are themselves plant traits measured in the same plots as grain yield, so the SHAP attributions describe statistical association within the trial rather than causal effects. Finally, the use of phenotypic data only limits direct modelling of the genetic basis of yield formation; integrating machine learning with genomic data can improve selection accuracy [11], so combined phenotypic–genomic approaches may yield more effective results in wheat breeding. Consistent with this constraint, the present study is presented as a single-site, single-season methodological proof of concept for the combined MGIDI + explainable-machine learning framework, and the genotype-specific selection outcomes should not be generalised to other environments without dedicated multi-environment confirmation. This limitation is reflected explicitly in the framing of the title, abstract and conclusions.

## 5. Conclusions

This study demonstrates the value of combining multi-trait selection with explainable machine learning for evaluating bread wheat genotypes under rainfed semi-arid conditions. The traits examined showed high heritability and a clear potential for genetic gain in yield components, and by ranking genotypes in a balanced way across multiple traits, MGIDI enabled the identification of superior genotypes that delivered a 9.50% increase in grain yield. The machine learning analyses indicated that the main determinants of grain yield were a limited number of key traits grain weight per spike, plant height and days to floweringand, supported by SHAP, these findings contribute to a better understanding of the biological basis of yield formation. Using MGIDI together with explainable machine learning allowed genotype selection and yield-determining traits to be evaluated simultaneously, providing a useful decision-support framework for breeding programmes. Because the results are based on a single environment, they should be validated in future multi-environment trials, and larger datasets and the integration of genomic information could further improve the accuracy and applicability of the approach. Given that all results are based on a single site and a single growing season, this study should be interpreted as a methodological demonstration whose selection outcomes require confirmation in multi-environment trials before being incorporated into formal cultivar recommendations.

## Figures and Tables

**Table 1:** Name, origin and type of the 20 bread wheat genotypes evaluated in this study.

No.	Genotype	Type/Origin
1	14STEMRRSN	CIMMYT advanced line
2	DZTB-1	Dicle University, Faculty of Agriculture
3	BEZOSTAYA	Maize Research Institute (Sakarya, Türkiye)
4	CEYHAN-99	Eastern Mediterranean Agricultural Research Institute (Adana, Türkiye)
5	DZTB-2	Dicle University, Faculty of Agriculture
6	DZTB-3	Dicle University, Faculty of Agriculture
7	EKİZ-43	Bahri Dağdaş International Agricultural Research Institute (Konya, Türkiye)
8	EMPİRE	Tekno Bil Tar Ar-Ge A.Ş. (Diyarbakır, Türkiye)
9	ENVOY	Australian-origin bread wheat (Australia)
10	HİLAR	Maize Research Institute (Sakarya, Türkiye)
11	DZTB-4	Dicle University, Faculty of Agriculture
12	DZTB-5	Dicle University, Faculty of Agriculture (advanced breeding line)
13	DZTB-6	Dicle University, Faculty of Agriculture
14	DZTB-7	Dicle University, Faculty of Agriculture
15	DZTB-8	Dicle University, Faculty of Agriculture
16	TOROS 1003	Toros Tarım Sanayi ve Ticaret A.Ş. (Türkiye)
17	Local-1	Regional landrace (Diyarbakır/Southeastern Anatolia)
18	Local-2	Regional landrace (Diyarbakır/Southeastern Anatolia)
19	Local-3	Regional landrace (Diyarbakır/Southeastern Anatolia)
20	Local-4	Regional landrace (Diyarbakır/Southeastern Anatolia)

**Table 2:** Physical and chemical properties of the experimental soil (0–30 cm layer, composite samples taken before sowing).

Parameter	Value	Unit
Saturation	63.20	%
pH (saturation paste)	8.15	–
Total salt	0.042	%
Electrical conductivity (EC)	1.030	dS m^−1^
Lime (CaCO_3_)	10.59	%
Organic matter (Walkley–Black)	0.77	%
Total nitrogen	0.04	%
Phosphorus (Olsen)	6.00	mg kg^−1^
Potassium (K)	493.26	mg kg^−1^
Calcium (Ca)	10,693.12	mg kg^−1^
Magnesium (Mg)	616.32	mg kg^−1^
Sodium (Na)	14.37	mg kg^−1^
Iron (Fe, DTPA)	8.86	mg kg^−1^
Zinc (Zn, DTPA)	0.29	mg kg^−1^
Manganese (Mn, DTPA)	23.10	mg kg^−1^
Copper (Cu, DTPA)	1.72	mg kg^−1^

**Table 3:** Grand mean, error CV%, broad-sense heritability (H^2^), genotypic (GCV%) and phenotypic (PCV%) coefficients of variation, expected genetic advance (GA) and genetic advance as a percentage of the mean (GAM%) at 25% selection intensity for the 14 traits evaluated.

Trait	Mean	CV%	H^2^	GCV%	PCV%	GA	GAM%	Class
Grain yield (kg da^−1^)	444.50	2.30	0.99	13.61	13.66	76.60	17.23	Moderate
Plant height (cm)	103.19	3.06	0.94	6.25	6.44	7.97	7.72	Low
Spike length (cm)	9.33	6.45	0.92	10.79	11.26	1.23	13.14	Moderate
Spikelets/spike	16.38	4.91	0.91	8.01	8.38	1.59	9.73	Low
Grains/spike	38.26	4.75	0.98	16.43	16.60	7.91	20.67	High
Grain weight/spike (g)	1.28	8.87	0.94	17.67	18.22	0.28	21.79	High
Days to heading (d)	144.45	1.10	0.94	2.13	2.20	3.78	2.62	Low
Days to flowering (d)	151.20	1.11	0.94	2.24	2.31	4.18	2.76	Low
NDVI (stem elongation)	0.77	2.75	0.84	3.12	3.41	0.03	3.62	Low
NDVI (heading)	0.70	2.81	0.90	4.29	4.52	0.04	5.18	Low
LAI (heading)	5.21	11.10	0.86	13.66	14.74	0.84	16.08	Moderate
SPAD (heading)	47.15	3.26	0.90	4.89	5.15	2.78	5.89	Low
Thousand-grain weight (g)	29.92	2.97	0.98	9.30	9.42	3.49	11.67	Moderate
Grain protein (%)	12.43	3.40	0.92	5.66	5.91	0.86	6.90	Low

GAM% classes follow the conventional thresholds: low, <10%; moderate, 10–20%; high, >20%.

**Table 4:** Varimax-rotated factor loadings for the 14 traits retained in the MGIDI analysis. Values in bold indicate the factor to which each trait was assigned.

Trait	FA1 (21.3%)	FA2 (21.3%)	FA3 (19.0%)	FA4 (14.8%)	FA5 (9.5%)
Grain yield (kg da^−1^)	−0.472	0.233	0.410	0.653	−0.045
Plant height (cm)	0.599	0.429	0.166	−0.107	−0.123
Spike length (cm)	0.035	0.009	0.921	0.067	0.113
Spikelets/spike	0.432	0.130	0.787	−0.037	0.187
Grains/spike	−0.034	−0.284	0.753	0.140	−0.334
Grain weight/spike (g)	−0.216	0.023	0.553	0.696	−0.174
Days to heading (d)	−0.980	−0.118	−0.059	0.077	−0.013
Days to flowering (d)	−0.990	−0.062	−0.063	0.052	−0.006
NDVI (stem elongation)	0.183	0.899	0.071	−0.091	−0.299
NDVI (heading)	0.147	0.822	0.022	−0.412	−0.110
LAI (heading)	−0.103	0.807	−0.088	0.323	−0.024
SPAD (heading)	0.006	−0.210	0.050	0.030	0.975
Thousand-grain weight (g)	0.051	−0.271	−0.096	0.894	0.119
Grain protein (%)	0.394	0.610	−0.314	−0.171	0.244

**Table 5:** Selection differential (SD) and selection gain (SG%, expressed in the agronomically desired direction) delivered by the five MGIDI-selected genotypes relative to the population mean.

Trait	Mean (All)	Mean (Selected)	SD	SG%
Grain yield (kg da^−1^)	444.50	486.75	42.24	9.50
Plant height (cm)	103.19	104.52	1.33	1.29
Spike length (cm)	9.33	9.91	0.58	6.23
Spikelets/spike	16.38	17.53	1.15	7.01
Grains/spike	38.26	40.05	1.78	4.66
Grain weight/spike (g)	1.28	1.45	0.17	12.88
Days to heading (d)	144.45	143.05	−1.40	0.97
Days to flowering (d)	151.20	149.50	−1.70	1.12
NDVI (stem elongation)	0.77	0.78	0.01	1.56
NDVI (heading)	0.70	0.70	−0.01	−0.87
LAI (heading)	5.21	5.70	0.49	9.31
SPAD (heading)	47.15	46.84	−0.31	−0.65
Thousand-grain weight (g)	29.92	30.24	0.32	1.08
Grain protein (%)	12.43	12.31	−0.12	−0.93

For days to heading and days to flowering the ideotype direction is earliness, so a negative SD corresponds to a positive (favourable) SG%.

**Table 6:** Block-stratified GroupKFold cross-validation performance of the five learners for grain-yield prediction.

Model	CV R^2^	RMSE (kg da^−1^)	MAE (kg da^−1^)
Gradient Boosting	0.740	30.55	24.43
XGBoost	0.703	32.63	25.87
Random Forest	0.658	35.04	27.78
ElasticNet	0.546	40.37	32.39
Ridge	0.526	41.26	32.90

**Table 7:** Convergence of permutation importance and mean |SHAP| attributions for the final Gradient Boosting model.

Trait	Permutation Δ (±SD)	Mean |SHAP|	SHAP Rank
Grain weight/spike	0.245 (±0.037)	16.36	1
Plant height	0.196 (±0.029)	13.60	2
Days to flowering	0.157 (±0.016)	11.84	3
Days to heading	0.063 (±0.013)	6.79	4
Thousand-grain weight	0.045 (±0.008)	6.40	5
Grains/spike	0.025 (±0.004)	4.41	6
LAI (heading)	0.021 (±0.004)	4.32	7
NDVI (stem elongation)	0.021 (±0.004)	4.24	8
Grain protein (%)	0.020 (±0.004)	2.83	9
Spike length	0.014 (±0.002)	2.46	10
Spikelets/spike	0.011 (±0.002)	2.27	11
SPAD (heading)	0.006 (±0.001)	1.36	12
NDVI (heading)	0.004 (±0.001)	0.96	13

## Data Availability

The datasets generated and analysed during the current study are available from the corresponding author on reasonable request.

## Supplementary Materials

The following supporting information can be downloaded at: https://www.mdpi.com/article/10.3390/plants15182787/s1, The Supplementary Materials include Table S1: Genotype means (±SEM) with LSD letter groupings, *LSD*_0.05_ and CV (%) for the 14 traits evaluated in the 20 bread wheat genotypes; different letters within a column indicate significant differences at *p* < 0.05 according to Fisher’s LSD test.
